# RWD insights into the global cost of Alzheimer’s and dementia

**DOI:** 10.1002/alz.089121

**Published:** 2025-01-09

**Authors:** Linus Jönsson

**Affiliations:** ^1^ Karolinska Institutet, Solna, Stockholm Sweden

## Abstract

It is well recognised that Alzheimer’s disease and related dementia disorders (ADRD) are associated with very high societal costs. The total global costs of dementia have been estimated to over 1.3 trillion US$ annually (Wimo, Seeher et al. 2023). Real‐world data (RWD) sources such as national patient registers, claims databases, disease and quality of care registers, offer an opportunity to assess resource utilisation and cost, as they reflect routine clinical practice in representative patient populations. However, as the majority of the costs of dementia fall outside of the health care system, most RWD sources offer only a partial view of the costs of dementia. Globally, only about 16% of costs are health‐care related, while 34% are related to social care and 50% are attributed to informal care (Wimo, Seeher et al. 2023). Most RWD sources specific for ADRD do not collect data on resource utilization or costs, though some offer opportunities for record linkage with databases containing such data. Therefore, most knowledge about costs of dementia has been derived from smaller observational studies with the specific purpose of assessing costs of care (Jonsson, Tate et al. 2023).

New methods for diagnosing and treating ADRD are being introduced, including amyloid‐targeting antibody therapies and blood biomarkers. RWD will play a key role in ensuring the appropriate and cost‐effective use of these technologies. To assess the cost implications of introducing these technologies in routine care, disease registries and other RWD sources need to be expanded to include key measures of resource utilization, including informal care and community care services.

